# The linkages of plant, litter and soil C:N:P stoichiometry and nutrient stock in different secondary mixed forest types in the Qinling Mountains, China

**DOI:** 10.7717/peerj.9274

**Published:** 2020-06-03

**Authors:** Yue Pang, Jing Tian, Xuan Zhao, Zhi Chao, Yuchao Wang, Xinping Zhang, Dexiang Wang

**Affiliations:** 1 College of Forestry, Northwest A&F University, Yangling, China; 2 Institute of Botany of Shaanxi Province, Xi’an, China; 3 Shaanxi Engineering Research Centre for Conservation and Utilization of Botanical Resources, Xi’an, China; 4 School of Art and Design, Xi’an University of Technology, Xi’an, China

**Keywords:** C:N:P stoichiometry, Nutrient stock, Multiple organs, Nutrient element correlation, Secondary mixed forest ecosystem

## Abstract

**Background:**

Carbon (C), nitrogen (N) and phosphorus (P) stoichiometric ratios are important indicators of ecosystem function and productivity. However, few studies have assessed the nutrient relationship between plant, litter and soil, and the nutrient stock in different secondary mixed forest types.

**Methods:**

We investigated the C, N and P concentrations and stoichiometric ratios in trees, understory plants, litter and soil layers in three different secondary mixed forest types (broadleaf mixed forests (BM), broadleaf-conifer mixed forests (BCM) and coniferous mixed forests (CM)) in the Qinling Mountains.

**Results:**

The results showed that significant differences in C:N:P stoichiometry were detected in multiple organs in the vegetation layers in the different forest types. Trees, shrubs and herbs all allocated more N and P in leaves and had a higher N:P ratio in leaves than in other organs. The C concentrations, C:N ratios and C:P ratios of all tree organs showed a decreasing order: BM < BCM < CM, while the N and P concentrations showed an increasing order: BM > BCM > CM. For litter and soil, BM had generally higher N and P concentrations than those of BCM and CM. The highest N and P stock was in tree branches-not in the stem, which had the highest biomass (except for P in CM). Compared with other forest types, CM stored more nutrients in the labile litter layer, while BM stored more nutrients in the stable soil layer. The net ecosystem nutrient element stock in BM was generally higher than that in BCM and CM. The C, N and P concentrations and stoichiometry in the plant organs, litter and soil were significantly correlated.

**Conclusion:**

Our findings demonstrate that nutrient concentrations in plant organs, litter and soil are tightly linked in secondary mixed forests.

## Introduction

Carbon (C), nitrogen (N) and phosphorus (P) are key elements for ecosystem organism construction and play vital roles in ecosystem processes (Chastain, Currie & Townsend, 2006; Song et al., 2014). Many studies have reported the C, N and P nutrient characteristics of vegetation, soil and litter in forest ecosystems (Cremer, Kern & Prietzel, 2016; Frédéric, Mathieu & Quentin, 2010; Inagaki, Miura & Kohzu, 2004). However, these studies independently studied the nutrient characteristics of different components of the ecosystem, ignoring the correlations between components.

Ecological stoichiometry, focusing on the interaction of chemical resources (elements) in the biogeochemical processes, has been regarded as a scientific and effective approach for exploring the feedbacks and relationships between the components in an ecosystem (Kennish, 2016). Previous studies have analyzed the C:N:P stoichiometric characteristics of plant organs, litter and soil at regional and global scales to reveal nutrient limitations of plants, nutrient cycling and feedback relationships (Han et al., 2005; Yang, Liu & An, 2018). These studies have advanced our understanding of ecosystem stoichiometric characteristics to some extent. However, for plant stoichiometry, these studies have mainly focused on certain organs, such as leaves and roots. Within different genetic characteristics and environmental factors, different plant organs play different functional roles, resulting in differences in nutrient concentrations among organs (Kerkhoff et al., 2006; Zhang et al., 2018c), and may further lead to nutrient characteristics differences of other components in the ecosystem. Therefore, it is important to quantify nutrient element stoichiometric variation in multiple plant organs and their nutrient relationships with other components in the ecosystem, which will provide further insights into nutrient cycling and ecological model building.

Plant nutrient concentrations and their ratios are generally influenced by forest types, as different habitat and nutrient conditions can affect the plant nutrient characteristics (Jerabkova, Prescott & Kishchuk, 2006). Han et al. (2005) reported that trees in deciduous forests had generally higher N and P concentration than these in coniferous forests. Further, these nutrient difference in vegetation will change the nutrient concentration of litter and soil (Capellesso et al., 2016), ultimately leading to the different nutrient stock of components among different ecosystems. Secondary forests account for 59.5% of the global forest cover and contain many forest types (Chokkalingam & De Jong, 2001; FAO, 2015). Although most previous studies have analyzed soil carbon stocks, stoichiometric and nutrient resorption and diverse ecological processes in secondary forests (Fonseca, Benayas & Alice, 2011; Kenzo et al., 2010; Zeng et al., 2017), the C, N and P nutrient patterns at the ecosystem level in different secondary mixed forest communities remain unclear. This insufficient knowledge might lead to the inaccurate estimation of secondary forest nutrient stock and underestimate the important role of secondary forests in the nutrient cycle (Attiwill & Adams, 1993; Mcdonald & Healey, 2000). Accordingly, exploring the characteristics of C, N and P nutrient concentrations and stock in different secondary mixed forests is urgently needed to meet the challenge of managing C and nutrient stocks worldwide.

Forests in the Qinling Mountains underwent from extensive logging during the 1960s and 1970s, which promoted the regeneration of diverse secondary forests. To advance natural forest resource protection and improve the ecological environment, the Chinese government initiated the “Natural Forest Protection Program” (Xu et al., 2006). Now, secondary forests account for 80% of the Qinling forest area, which has become an important secondary forest area in China (Chai et al., 2016). Previous studies have analyzed the structural characteristics of the community, soil nutrient characteristics, plant leaf C:N:P stoichiometry and microbial diversity among these secondary forests (Hou et al., 2018; Shi et al., 2019; Zhang et al., 2018b, Zheng et al., 2017). However, information about the relationship of C:N:P stoichiometry between vegetation, litter and soil and effects of different mixed forest types on ecosystem C:N:P stoichiometry and nutrient stock characteristics has rarely been evaluated.

In this study, we determined the C, N and P concentrations and stoichiometric ratios in trees, understory plants, litter and soil collected from three different secondary mixed forest types, namely, broadleaf mixed forests (BM), broadleaf-conifer mixed forests (BCM) and coniferous mixed forests (CM), in the Qinling Mountains. We hypothesized that the C, N and P stoichiometry and nutrient stock of different plant organs, litter and soil varied among different secondary mixed forest types due to differences in genetic characteristics and environmental factors. In addition, we predicted that the C, N and P concentrations in the plants, litter and soils might be highly coupled because of their cycling in the same system. Therefore, the objectives of this research were to: (1) examine the C, N and P concentrations and stoichiometric characteristic differences of multiple plant organs, litter and soil among different secondary mixed forest types; (2) quantify the nutrient stock capacity of the C, N and P elements in different secondary mixed ecosystems; and (3) explore the relationships of C:N:P stoichiometry between the plant, litter and soil.

## Materials and Methods

### Study site description

The field research was conducted at the Qinling National Forest Ecosystem Research Station (Huoditang Experimental Forest Farm of Northwest A&F University) in Ningshaan County (33°18′–33°28′N, 108°21′–108°39′E), Shaanxi Province, China. The landform of the station is characterized by an abrupt and broken landscape, with altitudes ranging from 800 to 2,500 m and a mean slope of approximately 35°. The soil in this area is composed of Cambisols, Umbrisols and Podzols (FAO), and the mean soil depth is 50 cm (Yu et al., 2013). This region has a subtropical humid montane climate, with an average annual precipitation of 1,000 mm. Over 50% of the precipitation falls from July to September, and the average annual humidity is approximately 77%. The average temperature is 10.5 °C, with an extreme minimum temperature of −9.5 °C and an extreme maximum temperature of 35 °C. The plant growth period is approximately 177 days, and the average frost-free period is approximately 199 days (Delian, 2004). The forest farm covers an area of 22.25 km^2^. The forests had been rotated felling or experienced firewood cutting between 1976 and 1978 in the Huoditang Experimental Forest Farm, and much of the area is now covered by secondary growth. Currently, the main tree species in this area are *Ouercus aliena var. auteserrata*, *Quercus variabilis*, *Pinus armandii*, *Betula albosinensis*, *Picea asperata*, *Populus davidiana* and other broadleaf species. Based on the vegetation deforestation history and restoration status, three secondary mixed forest types (BM, BCM and CM) were selected. Detailed information about each secondary mixed forest type is presented in Table 1.

**Table 1:** Characteristics of sample plots in three secondary mixed forests.

Forest types	BM	BCM	CM
Altitude (m)	1,900–2150	2,000–2,100	1,800–2,000
Slope aspect	Northwest	Northeast	Northwest
Slope position	Central	Central	Below
Slope gradient (°)	16–24	11–20	15–22
Fertigation	No	No	No
Trees	*Betula albosinensis*	*Pinus armandii*	*Pinus armandii*
	*Quercus L*.	*Quercus L*.	*Picea asperata*
	*Acer davidii*		
Shrubs	*Schisandra sphenanthera*	*Schisandra sphenanthera*	*Viburnum betulifolium*
	*Viburnum betulifolium*	*Smilax china*	*Lonicera fragrantissima*
	*Rubus mesogaeus*	*Viburnum betulifolium*	*Rubus mesogaeus*
Herbaceous	*Matteuccia intermedia*	*Tripterospermum chinense*	*Athyrium sinense*
	*Lysimachia christinae*	*Viola verecunda*	*Tripterospermum chinense*
	*Carex duriuscula*	*Carex duriuscula*	*Carex duriuscula*
DBH (cm)	17.24 ± 1.76	13.98 ± 0.74	19.06 ± 0.52
Height (m)	10.85 ± 0.22	11.84 ± 0.56	19.79 ± 0.34
Density (n ha^−1^)	933 ± 246	1,333 ± 30	783 ± 88

**Note:**

BM, broadleaf mixed forests; BCM, broadleaf-conifer mixed forests; CM, coniferous mixed forests; DBH, diameter at breast height.

### Experimental design

**Figure 1:**
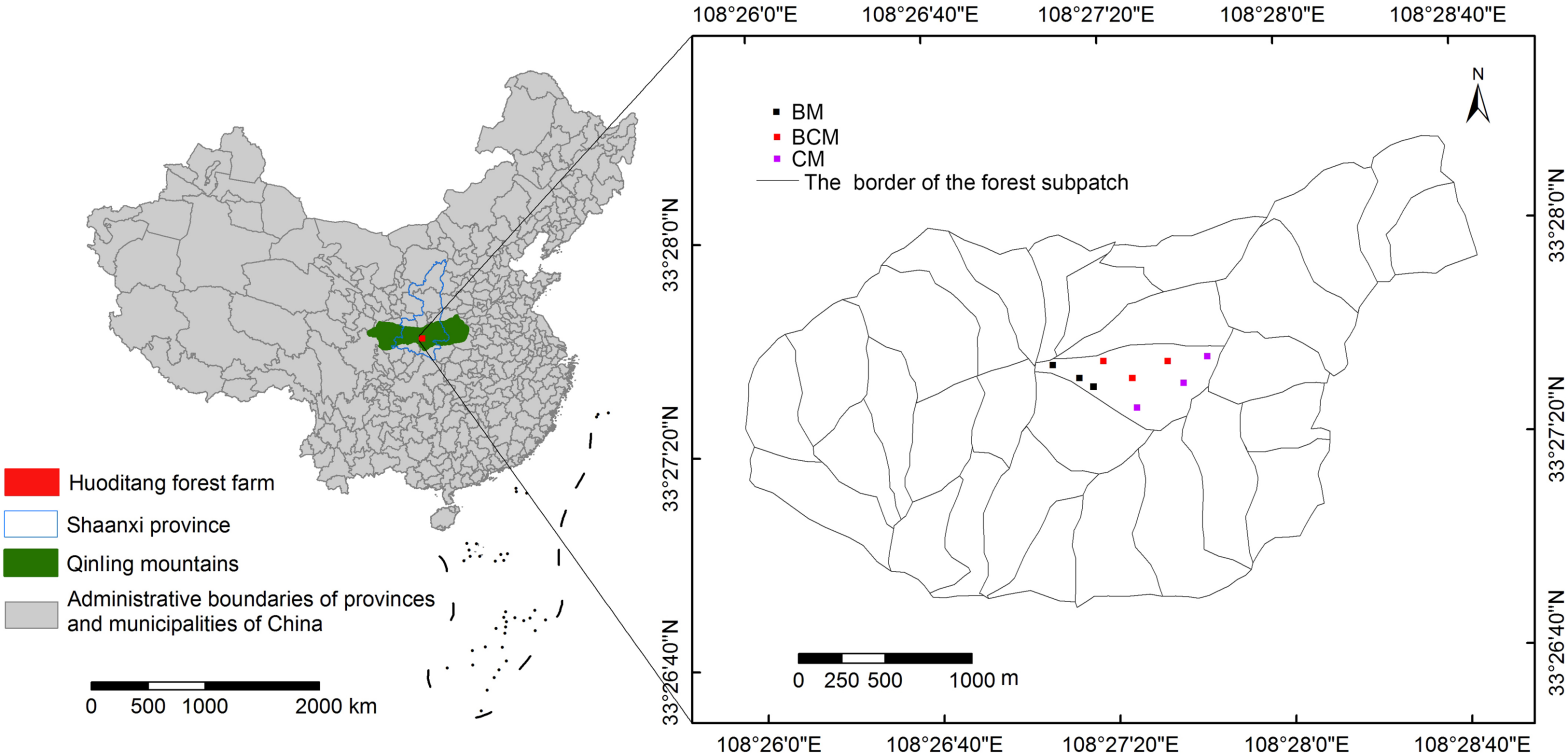
Geographic location of the Huoditang experimental forest farm and the sampling plots. BM, broadleaf mixed forests; BCM, broadleaf-conifer mixed forests; CM, coniferous mixed forests.

The study was conducted from July to August 2017 at the Huoditang Experimental Forest Farm. All selected sites were located on similar slopes, aspects, slope gradients and elevations. Each secondary mixed forest type was represented by three independent replicate sites, and the space between any two sites was large enough to exclude spatial dependance for the soil variables. Three replicate plots (20 × 20 m) were randomly established at each site for the subsequent plant, litter and soil sampling (Fig. 1). For each plant, litter and soil variable, the average value of the three replicated plots was taken as the observation for the whole site. Finally, in total, nine observations were established (three different secondary mixed forest types × three replicate sites) for each variable.

### Plant, litter and soil investigation and sampling

The diameter at breast height (DBH ≥ 5 cm, 1.3 m) of all trees in each plot was measured, and the trees were also classified and counted by species. After that, different organ samples of trees were obtained by species. Mature and healthy leaves were picked from the east, west, south and north directions of the tree crown and branches (diameter <1 cm) were cut form the upper, middle and lower parts of the canopies. The machete and increment bore were used to obtain the bark and stem samples, respectively, at the DBH location. Roots (diameter < 1 cm) were dug up from the 0 to 60 cm soil layer, and root samples were collected by removing the surrounding soil along a main root of a specific plant species until the roots appeared. These organ samples from the trees were oven dried at 70 °C to constant weight. Based on the DBH and tree height, the biomass of the components (leaves, branches, stems, bark and roots) of each tree species in the three secondary mixed forest plots was calculated using published species-specific allometric equations developed for trees within or near the study area (Table S1). To better reflect the relative contributions of multiple tree species at the plot level, we first calculated the biomass of the organs of the corresponding species according to the allometric growth equation and obtained the contribution ratio of the organs of different species. Then the different organ samples of the tree were mixed according to the ratios.

Shrub and herb biomass were determined using total harvesting destructive sampling techniques. Five shrub subplots (2 × 2 m) and five herb subplots (1 × 1 m) were established along the diagonals of each plot for sample collection. Shrub plants were separated into leaves, stems and roots, and herbs were separated into aboveground and belowground components. For litter sampling, all organic material within five 1 × 1 m subplots was collected from each plot. There were no corresponding allometric equations for shrubs and herbs in the study area, and the same components of shrubs, herbs and litter were mixed uniformly into one sample. Finally, the subsamples of shrub, herb and litter were transported to the laboratory and oven dried at 70 °C to a constant weight.

For soil sampling, nine replicate sampling points were established along an “S” shape in nine plots. After removing the litter layer and biological crusts, nine soil samples at 0–20, 20–40 and 40–60 cm were obtained separately from each point using a soil auger (40-mm inner diameter) and were fully homogenized to form one composite soil sample for each soil layer in each plot. The plant roots, fauna and debris were removed by hand, and the gravel (rock fragments >2 mm) was reserved to measure the percentage of stones. The remaining soil samples were sieved (<2 mm) and air dried at room temperature for chemical property analysis. Soil bulk density (BD) samples were obtained randomly from three points per plot by volumetric rings (100 cm^3^). The nutrient element stock of C, N and P in each soil layer was calculated using the following equation:

$${{S}_n} = {C_n} \times {\rm BD}_{n} \times {L_n} \times {10^{ - 1}}$$

Where *S_n_* is the C, N and P stock of soil in the *n*-th soil layer (t·ha^−1^); *C_n_*, BD*_n_*, and *L_n_* are the C concentration (mg·g^−1^), soil bulk density (g·cm^−3^), and soil depth (cm) of the *n*-th soil layer, respectively; and 10^−1^ is the unit conversion factor.

### Plant, litter and soil physicochemical measurements

The C, N and P concentrations in the tree, shrub and herb organs and litter were analyzed after the samples were ground into a powder with a plant-sample mill (1,093 Sample Mill, Hoganas, Sweden). The organic carbon (OC) contents of the plant, litter and soil samples were measured using the K_2_Cr_2_O_7_ oxidation method (Bao, 2000). The total nitrogen (TN) and total phosphorus (TP) concentrations of the plant, litter and soil samples were determined by colorimetric method with an automatic discontinuous elemental analyzer (Clever chem200+, Germany) after digestion with H_2_SO_4_ and H_2_O_2_. The volume of gravel (rock fragments >2 mm) was measured using the drainage method. The soil BD was determined using the soil core method and obtained by calculating the ratio of soil mass to total volume (g·cm^−3^) after oven dried at 105 °C to a constant weight (De Vos et al., 2005).

### Data analyses

The total ecosystem C, N and P stock values were based on the combination of trees, shrubs, herbs, litter and soil pool. The mean and standard error of the investigated variables (e.g., C, N and P concentrations, C, N and P stocks, C:N, C:P and N:P ratios) of plant organs, litter and soil mixtures were calculated for each organ, site and soil depth separately. Data were checked for normality and homogeneity of variance and, if necessary, were transformed. The effects of organ, soil layer and forest type on the concentration, stoichiometry and stocks of the nutrient elements (C, N and P) were tested using one-way ANOVA and least significant difference (LSD) multiple comparison (*p* < 0.05). The Pearson correlation was used to determine the relationships of C:N:P stoichiometry between plant, litter and soil. All statistical analyses were performed using R version 3.5.0 (R Development Core Team, 2018).

## Results

### Plant and litter biomass and soil bulk density

The biomass of plant organs was generally different among different organs and forest types (Figs. S1A and S1B). For total plant biomass (Table S2), the shrub total biomass in BCM (4.15 t·ha^−1^) was significantly higher than that in CM (2.26 t·ha^−1^), and there were no significant differences between BCM and BM (3.3 t·ha^−1^). The herb total biomass in CM (1.08 t·ha^−1^) was significantly higher than that in both BM (0.55 t·ha^−1^) and BCM (0.66 t·ha^−1^). Although the tree total biomass was nonsignificant among the three forest types, it accounted for more than 96% of the ecosystem total plant biomass in all forest types. In addition, the litter biomass in CM (5.52 t·ha^−1^) was significantly higher than that in BM (3.86 t·ha^−1^) and BCM (4.21 t·ha^−1^) (Table S2). For organ biomass (Figs. S1A and S1B), the highest biomass occurred in the stem for tree, root for shrub and aboveground portion for herb, ranging from 61.94 to 83.74 t·ha^−1^, 1.50 to 1.88 t·ha^−1^, 0.24 to 0.46 t·ha^−1^, respectively. Inconsistent biomass of plant organs was observed in vegetation layers among different forest types; however, it was nonsignificant.

Only in the BM was the soil BD of the 0–20 cm soil layer significantly lower than that of the 40–60 cm soil layer, although it was not statistically significant among the different soil layers in the BCM and CM (Fig. S1C). There was no significant difference in soil BD at the same soil layer between different forest types (Fig. S1C).

### C:N:P stoichiometric characteristics in ecosystem components

**Figure 2:**
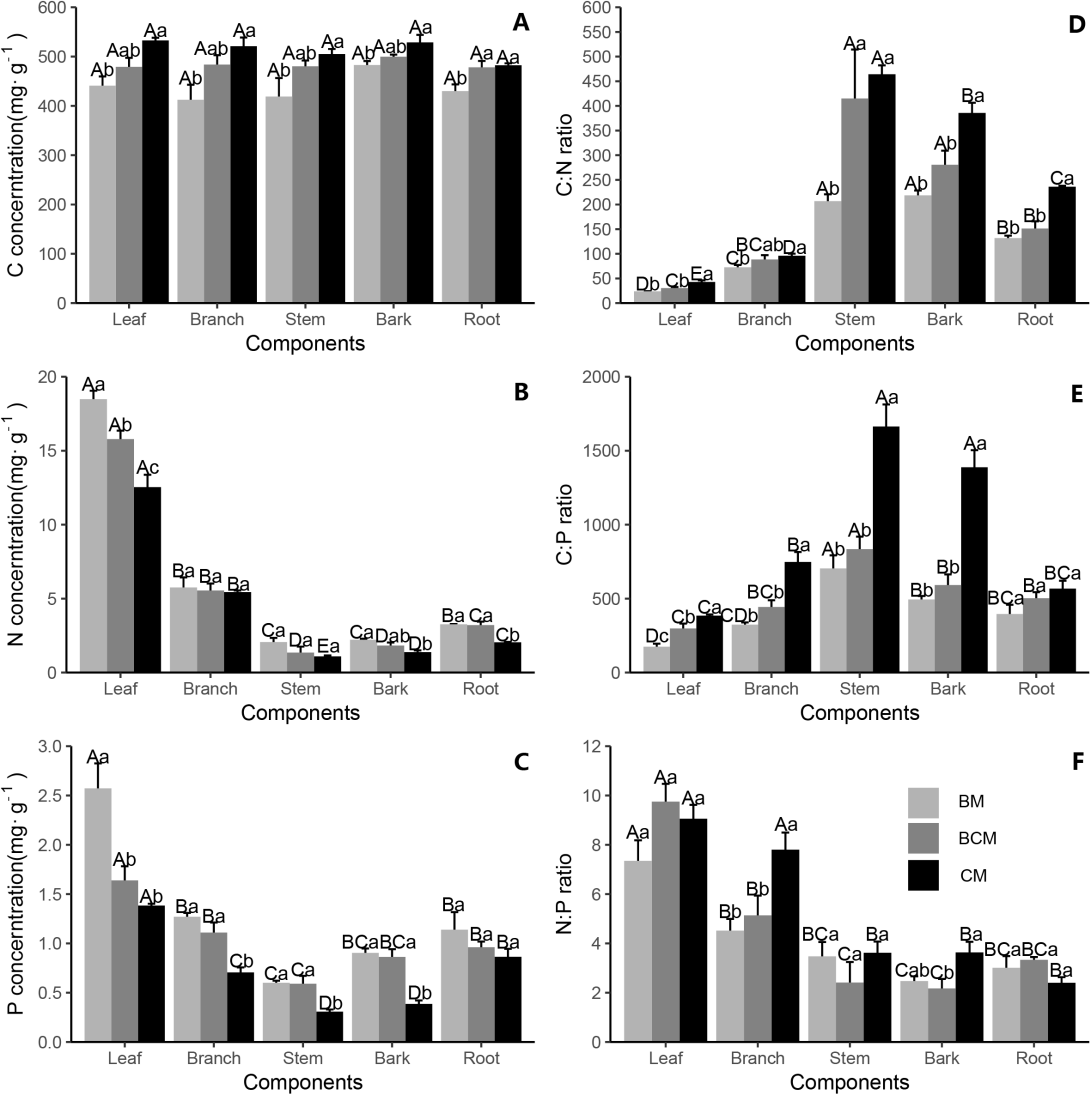
Stoichiometric characteristics of tree organ C, N and P in three secondary mixed forests. (A, B, C) showed the concentration of carbon, nitrogen and phosphorus in tree organs; (D, E, F) showed the stoichiometric ratios of C:N, C:P and N:P in tree organs. Different lowercase letters above the bars indicate significant differences among different forest types for the same organ (*p* < 0.05), while different uppercase letters indicate significant differences among different organs for the same forest type (*p* < 0.05). BM, broadleaf mixed forests; BCM, broadleaf-conifer mixed forests; CM, coniferous mixed forests.

The stoichiometry varied greatly in different plant organs, litter and soil layers under different forest types. In the tree layer, the C concentration of all organs in CM was notably higher than that in BM, while it was similar with BCM (Fig. 2A). The C concentration was nonsignificant between different organs for all forest types (Fig. 2A). Among tree organs, the leaves and stem had significantly higher and lower N and P concentrations than the other organs in all forest types together (Figs. 2B and 2C). The N and P concentrations in all tree organs had the same pattern among the different forest types, showing the increasing order of BM > BCM > CM (Figs. 2B and 2C). Leaves and stems had the lowest and highest ratios of C:N and C:P for all forest types, respectively, showing a decreasing order of BM < BCM < CM (Figs. 2D and 2E). The N:P ratio in leaves was notably higher than that in other organs among all forest types (except leaves and branches in CM) (Fig. 2F). The N:P ratios of branches and bark in CM were significantly higher than those of the other two forest types, while the values were typically nonsignificant in other organs in all forest types (Fig. 2F).

**Figure 3:**
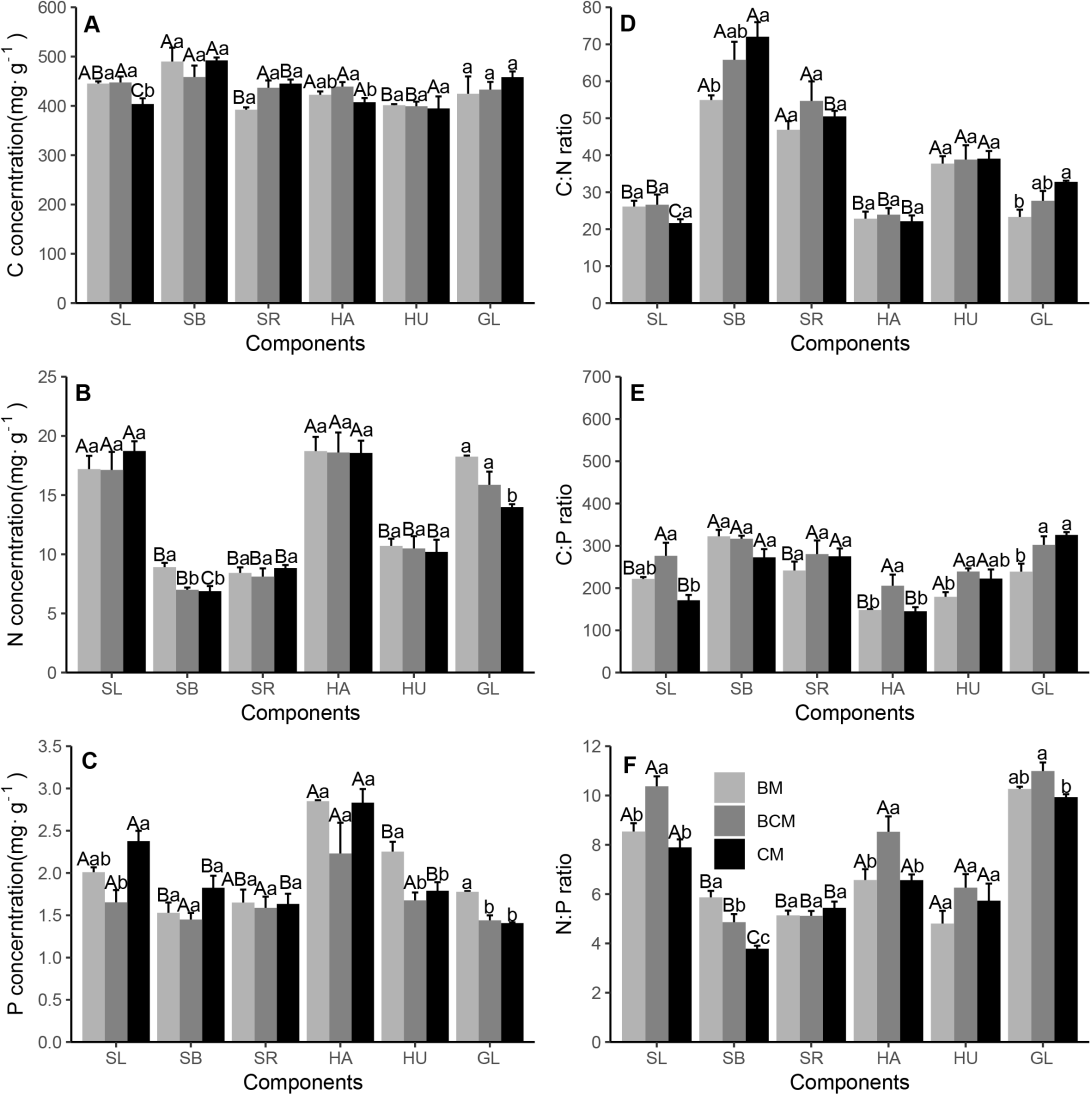
Stoichiometric characteristics of shrub and herb organs and litter layer C, N and P in three secondary mixed forests. (A, B, C) showed the concentration of carbon, nitrogen and phosphorus in shrub and herb organs and litter layer; (D, E, F) showed the stoichiometric ratios of C:N, C:P and N:P in shrub and herb organs and litter layer. Different lowercase letters above the bars indicate significant differences among different forest types for the same organ (*p* < 0.05), while different uppercase letters indicate significant differences among different organs for the same forest type (*p* < 0.05). SL, shrub leaf; SB, shrub branch; SR, shrub root; HA, herb aboveground; HU, herb underground; GL, ground litter. BM, broadleaf mixed forests; BCM, broadleaf-conifer mixed forests; CM, coniferous mixed forests.

In the shrub layer, the highest C concentration was observed in the branches for the three forest types, while the highest N and P concentrations were in the leaves (Figs. 3A–3C). Leaves in CM had significant lower and higher C and P than BM, while branches in BM had significant higher N than the other two forests. Shrub branches and leaves had the highest and lowest ratios of C:N and C:P for all forest types, while the highest N:P ratio was observed in leaves (Figs. 3D–3F). Branches in BM had significant lower and higher C:N and N:P than CM, while leaves in BCM had significant higher C:P and N:P than the other two forests. In the herb layer, the aboveground leaf C, N and P concentrations were significantly higher than those in the underground root, while the aboveground leaf C:N and C:P ratios were significantly lower than those in underground root (except for C:P in BCM) (Figs. 3A–3E). Leaves in BCM had significant higher C and C:P than the other two forests, while roots in BM had significant higher and lower P and C:P. Although the herb N:P ratio was nonsignificant among different organs, it was generally higher in leaves than in roots (Fig. 3F). Leaves in BCM had significant higher N:P than the other two forests. In the litter layer, the C:N:P stoichiometric characteristics were similar to the results of the tree layer (except for N:P). The C concentration was generally higher in CM than in BM and BCM (Fig. 3A). The N and P concentrations in BM were significantly higher than those in BCM and CM, while the opposite trend was observed, that is, the C:N and C:P ratios were significantly lower in BM than in BCM and CM (Figs. 3B–3E). BM and BCM had relatively higher N:P ratios than CM (Fig. 3F).

**Figure 4:**
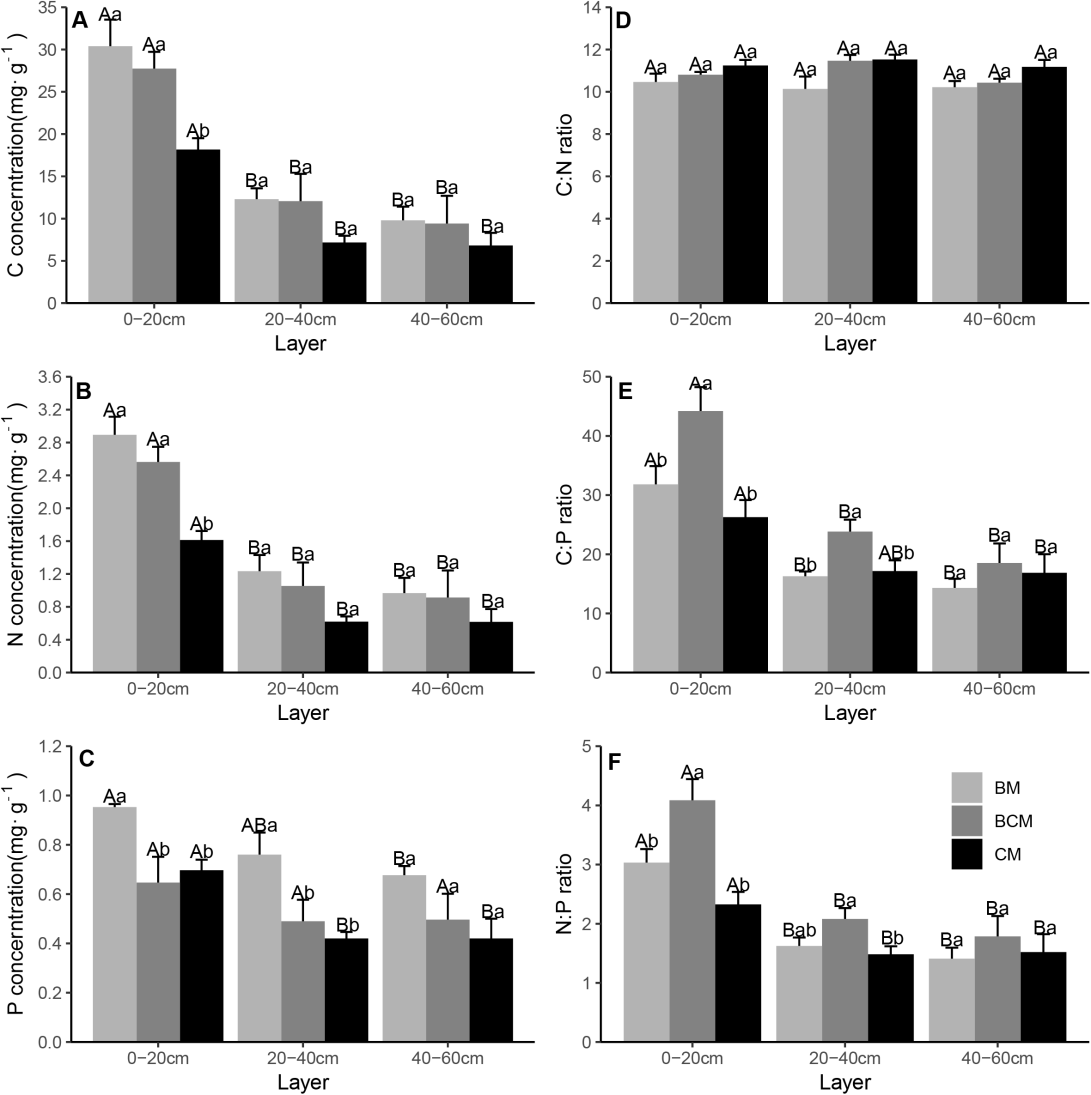
Stoichiometric characteristics of soil layer C, N and P in three secondary mixed forests. (A, B, C) showed the concentration of carbon, nitrogen and phosphorus in soil layers; (D, E, F) showed the stoichiometric ratios of C:N, C:P and N:P in soil layers. Different lowercase letters above the bars indicate significant differences among different forest types for the same soil layer (*p* < 0.05), while different uppercase letters indicate significant differences among different soil layers for the same forest type (*p* < 0.05). BM, broadleaf mixed forests; BCM, broadleaf-conifer mixed forests; CM, coniferous mixed forests.

For the soil level, the concentrations of C, N and P and the ratios of C:P and N:P in topsoil (0–20 cm) were significantly higher than those in subsoil (20–40 and 40–60 cm), while the C:N ratio was nonsignificant among different soil layers (Figs. 4A–4F). The C and N concentrations in CM were significantly lower than those in BCM and BM only in topsoil, while the P concentration was significantly higher in the 0–20 and 20–40 cm soil layers in BM than in BCM and CM (Figs. 4A–4C). The C:P and N:P ratios in the 0–20 and 20–40 cm soil layers in BCM were significantly higher than those in BM and CM, while the C:N ratio was nonsignificant among the different forest types (Figs. 4D–4F).

### C, N and P nutrient stock in ecosystem

**Figure 5:**
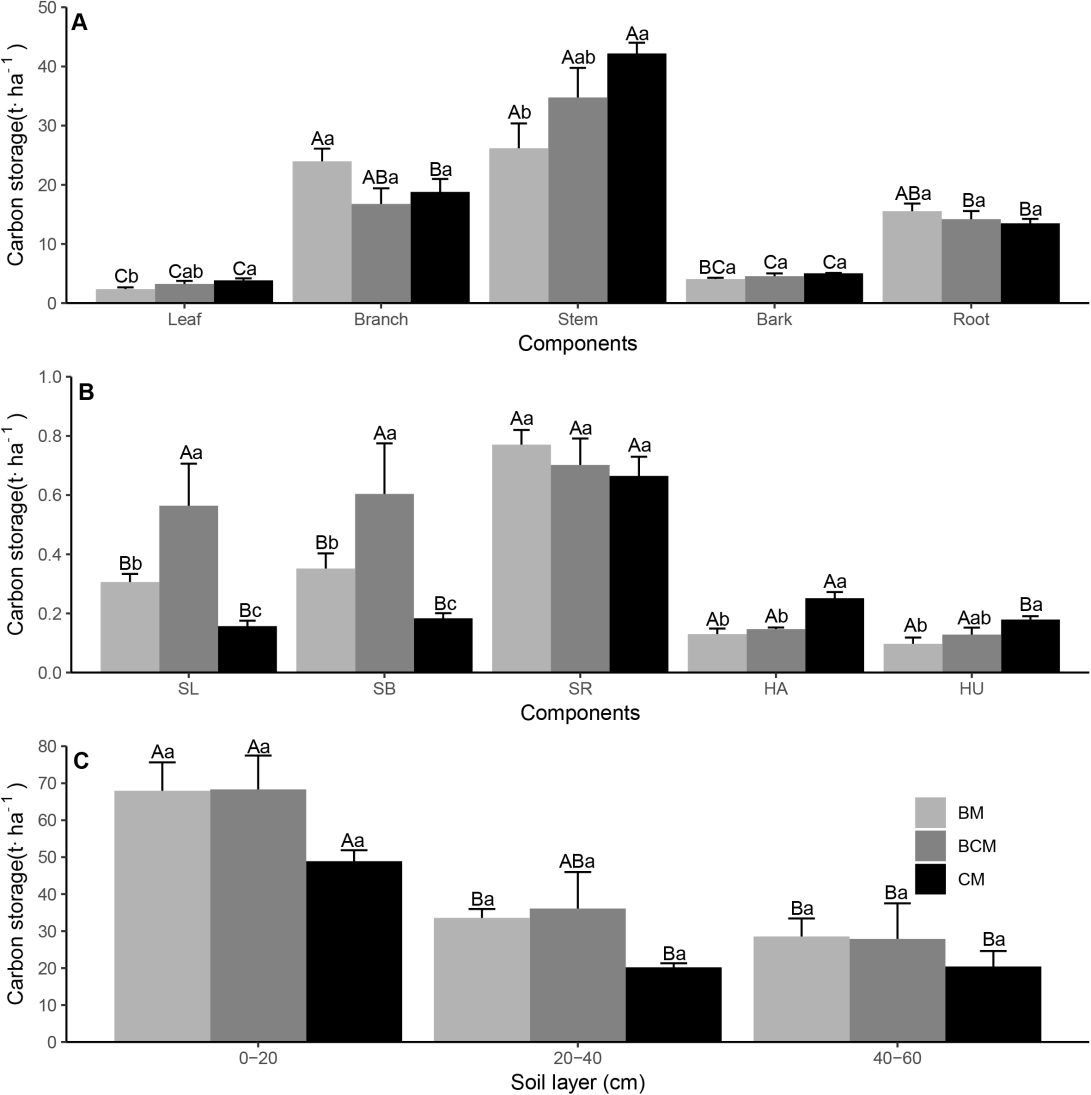
Carbon storage of trees (A), understory plants (B) organs and soil layers (C) in three secondary mixed forests. Different lowercase letters above the bars indicate significant differences among different forest types for the same organ or soil layer (*p* < 0.05), while different uppercase letters indicate significant differences among different organs or soil layers for the same forest type (*p* < 0.05). SL, shrub leaf; SB, shrub branch; SR, shrub root; HA, herb aboveground; HU, herb underground. BM, broadleaf mixed forests; BCM, broadleaf-conifer mixed forests; CM, coniferous mixed forests.

**Figure 6:**
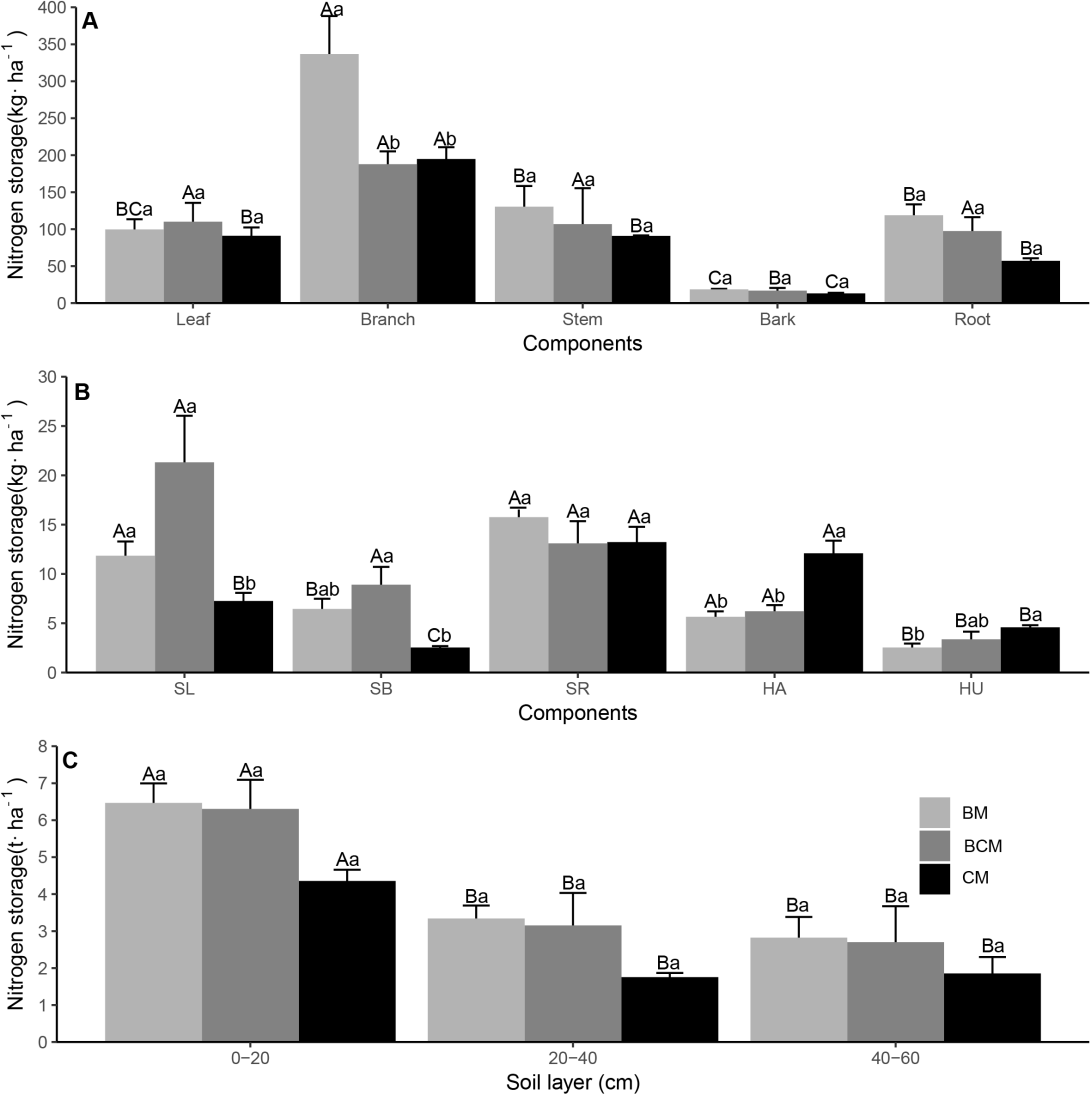
Nitrogen storage of trees (A), understory plants (B) organs and soil layers (C) in three secondary mixed forests. Different lowercase letters above the bars indicate significant differences among different forest types for the same organ or soil layer (*p* < 0.05), while different uppercase letters indicate significant differences among different organs or soil layers for the same forest type (*p* < 0.05). SL, shrub leaf; SB, shrub branch; SR, shrub root; HA, herb aboveground; HU, herb underground. BM, broadleaf mixed forests; BCM, broadleaf-conifer mixed forests; CM, coniferous mixed forests.

**Figure 7:**
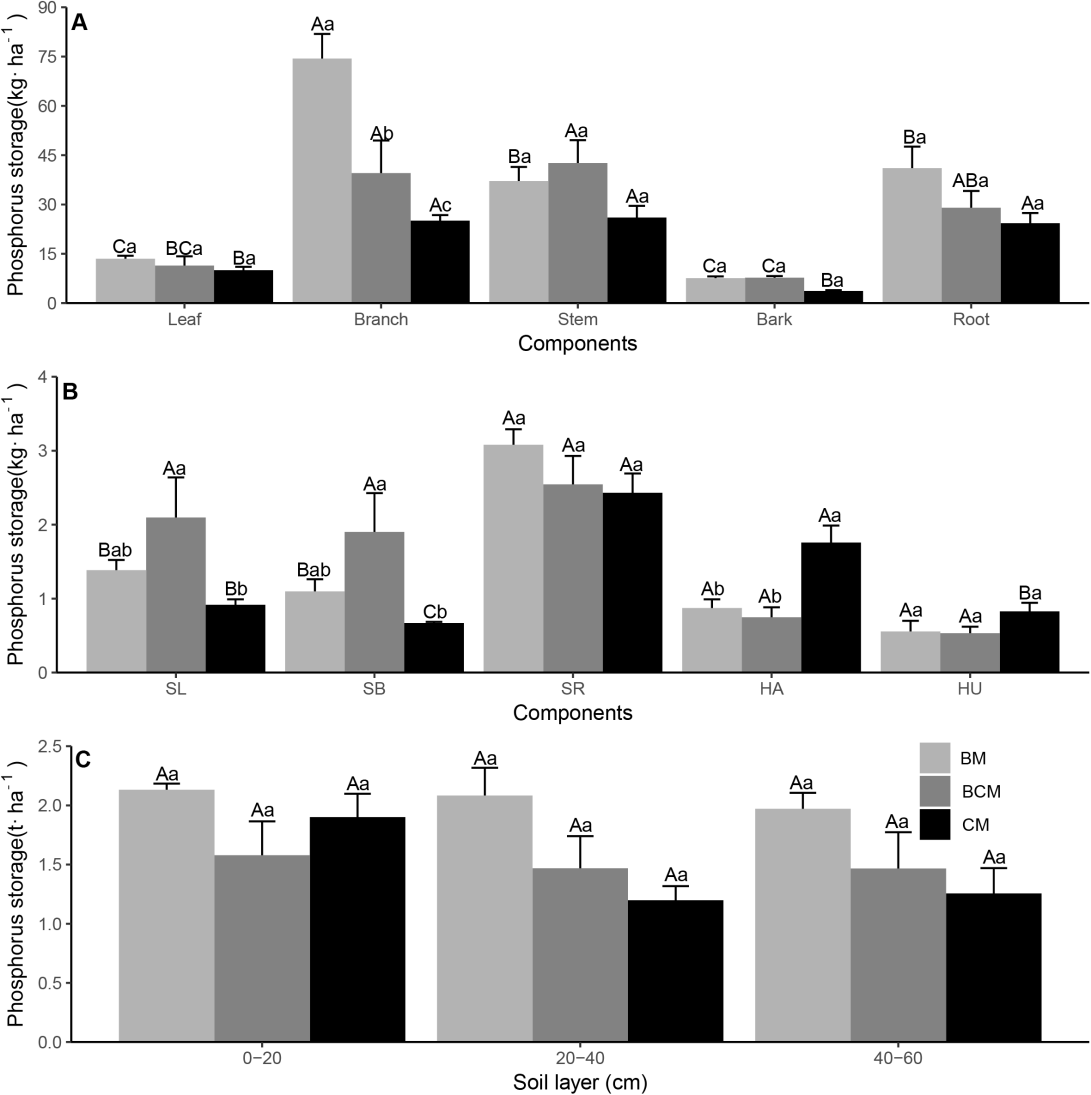
Phosphorus storage of trees (A), understory plants (B) organs and soil layers (C) in three secondary mixed forests. Different lowercase letters above the bars indicate significant differences among different forest types for the same organ or soil layer (*p* < 0.05), while different uppercase letters indicate significant differences among different organs or soil layers for the same forest type (*p* < 0.05). SL, shrub leaf; SB, shrub branch; SR, shrub root, HA, herb aboveground; HU, herb underground. BM, broadleaf mixed forests; BCM, broadleaf-conifer mixed forests; CM, coniferous mixed forests.

The C, N and P nutrient stock varied greatly in the different plant organs, litter and soil layers in the different forest types (Figs. 5–7; Tables 2–4). For the plant layer, the highest C stock was observed in the stems of trees, roots of shrubs and leaves of herbs, while the highest N and P stock was generally observed in branches of trees (except for P in BCM and CM), roots of shrubs (except for N in BCM) and leaves of herbs (Figs. 5–7A and 7B). The C stock of tree stems in CM was significantly higher than that in BM, while the N and P stock values of tree branches in BM were significantly higher than those in BCM and CM (Figs. 5–7A). Additionally, the C, N and P stock values of shrub leaves and branches in BCM were generally higher than those in the other two forest types, while the C, N and P stock values of herb leaves in BM and BCM were considerably lower than those in CM (Figs. 5–7B). Regarding nutrient element stock of total plant biomass, the C, N and P stock values of shrub biomass in BCM were generally higher than those in BM and CM, while the C, N and P stock values of herb biomass in CM were significantly higher than those in BM and BCM (Tables 2–4). The nutrient element stock of total tree biomass had the highest percentage among the plant layer, and the P stock of total tree biomass in BM was significantly higher than that in CM (Tables 2–4). For the litter layer, the share of C, N and P stored in litter biomass in CM generally exceeded that in BM and BCM (Tables 2–4).

**Table 2:** Carbon storage of plant total biomass, litter total biomass, soil and net ecosystem. Different letters indicate significant differences (*p* < 0.05) among forest types based on a one-way ANOVA followed by an LSD test.

Ecosystem pool	BM		BCM		CM	
	C storage	Percentage	C storage	Percentage	C storage	Percentage
Tree (t ha^−1^)	72.09 ± 4.82	35.09	73.49 ± 9.71	35.03	83.35 ± 3.11	47.13
Shrub (t ha^−1^)	1.43 ± 0.06ab	0.70	1.87 ± 0.26a	0.89	1.00 ± 0.07b	0.57
Herb (t ha^−1^)	0.23 ± 0.01b	0.11	0.27 ± 0.01b	0.13	0.43 ± 0.03a	0.24
G-litter (t ha^−1^)	1.63 ± 0.16b	0.80	1.82 ± 0.10b	0.87	2.53 ± 0.16a	1.43
Soil (t ha^−1^)	130.05 ± 13	63.30	132.30 ± 25	63.08	89.54 ± 4.01	50.63
Net ecosystem (t ha^−1^)	205.43 ± 10	100	209.75 ± 35	100	176.86 ± 7.14	100

**Note:**

BM, broadleaf mixed forests; BCM, broadleaf-conifer mixed forests; CM, coniferous mixed forests.

**Table 3:** Nitrogen storage of plant total biomass, litter total biomass, soil and net ecosystem. Different letters indicate significant differences (*p* < 0.05) among forest types based on a one-way ANOVA followed by an LSD test.

Ecosystem pool	BM		BCM		CM	
	N storage	Percentage	N storage	Percentage	N storage	Percentage
Tree (kg ha^−1^)	704.16 ± 41	5.24	518.96 ± 112	4.05	446.93 ± 24	5.24
Shrub (kg ha^−1^)	34.06 ± 0.70a	0.25	43.33 ± 6.7a	0.34	23.03 ± 1.05b	0.27
Herb (kg ha^−1^)	8.20 ± 0.16b	0.06	9.59 ± 0.77b	0.07	16.68 ± 1.5a	0.20
G-litter (kg ha^−1^)	70.35 ± 5.07	0.52	66.42 ± 2.89	0.52	77.27 ± 4.51	0.91
Soil (t ha^−1^)	12.63 ± 1.33	93.93	12.16 ± 2.39	95.02	7.97 ± 0.29	93.43
Net ecosystem (t ha^−1^)	13.45 ± 1.32	100	12.80 ± 2.5	100	8.53 ± 0.32	100

**Note:**

BM, broadleaf mixed forests; BCM, broadleaf-conifer mixed forests; CM, coniferous mixed forests.

**Table 4:** Phosphorus storage of plant total biomass, litter total biomass, soil and net ecosystem. Different letters indicate significant differences (*p* < 0.05) among forest types based on a one-way ANOVA followed by an LSD test.

Ecosystem pool	BM		BCM		CM	
	P storage	Percentage	P storage	Percentage	P storage	Percentage
Tree (kg ha^−1^)	173.67 ± 13a	2.72	130.27 ± 23ab	2.8	89.13 ± 3.9b	2.00
Shrub (kg ha^−1^)	5.56 ± 0.19ab	0.09	6.54 ± 1.04a	0.14	4.01 ± 0.2b	0.09
Herb (kg ha^−1^)	1.43 ± 0.04b	0.02	1.28 ± 0.07b	0.03	2.58 ± 0.32a	0.06
G-litter (kg ha^−1^)	6.86 ± 0.53ab	0.11	6.04 ± 0.07b	0.13	7.77 ± 0.36a	0.17
Soil (t ha^−1^)	6.19 ± 0.39	97.06	4.51 ± 0.8	96.9	4.36 ± 0.35	97.76
Net ecosystem (t ha^−1^)	6.37 ± 0.39	100	4.66 ± 0.84	100	4.46 ± 0.34	100

**Note:**

BM, broadleaf mixed forests; BCM, broadleaf-conifer mixed forests; CM, coniferous mixed forests.

For the soil layer, the stock of C and N in the mineral topsoil (0–20 cm) was significantly higher than that in the subsoil (20–60 cm), while the P stock was nonsignificant among the different soil layers (Figs. 5–7C). Although there was no notable difference in nutrient element stock at the same soil layer among different forest types, the nutrient element stock in BM was generally higher than that in BCM and CM (Figs. 5–7C). Regarding the total soil nutrient element stock and net ecosystem nutrient element stock, the C, N and P stock values were all nonsignificant under the three forest types (Tables 2–4). However, the soil layer had the highest nutrient element stock among different ecosystem components, and both the total soil nutrient element stock and the net ecosystem nutrient element stock in BM were generally higher than those in BCM and CM (Tables 2–4).

### Connections of C:N:P stoichiometric among ecosystem components

**Figure 8:**
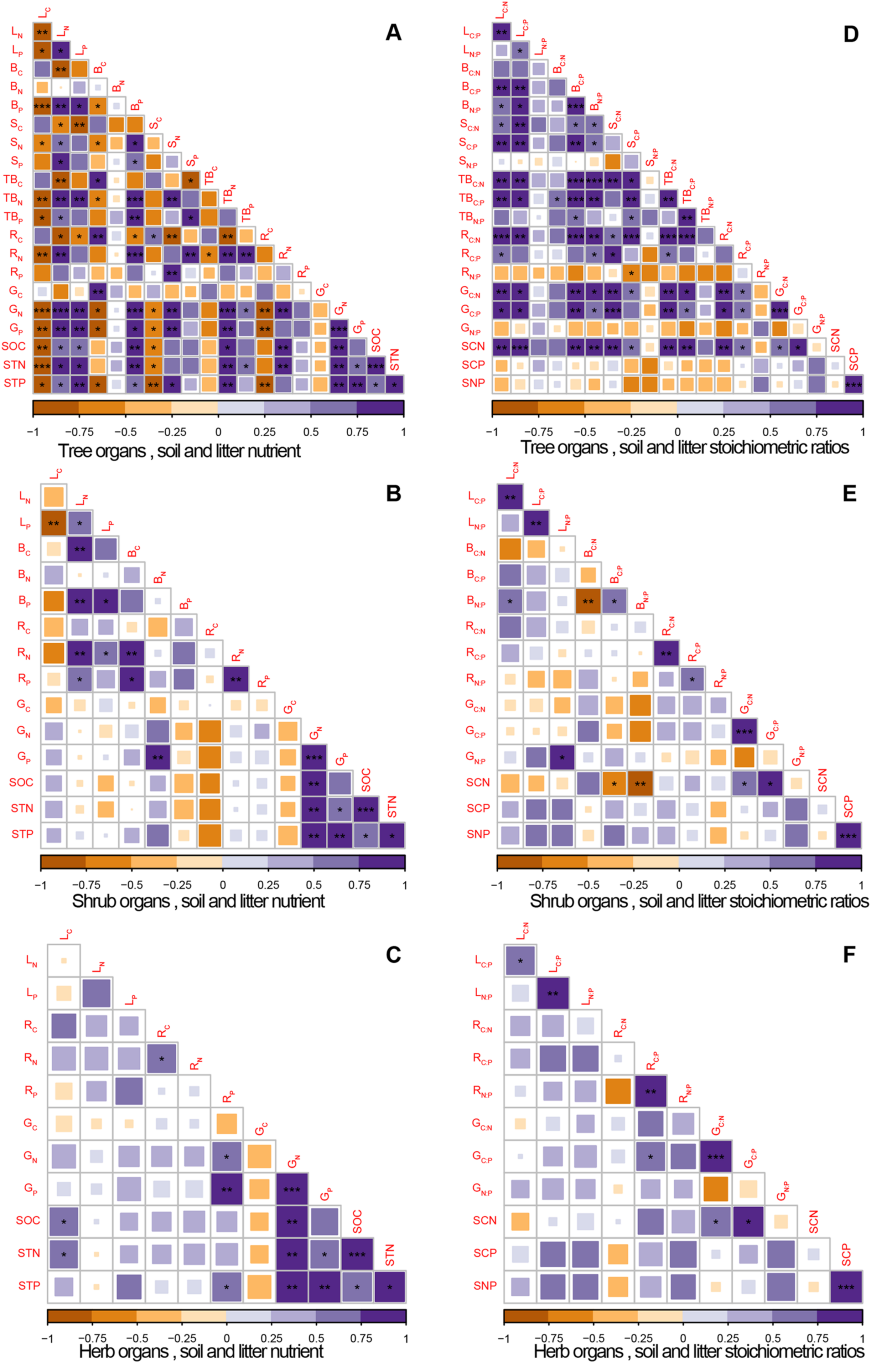
Pearson’s correlation matrix between plant organs, litter and soil C, N and P concentrations and stoichiometric ratios. (A, B, C) showed the correlation between the concentration of carbon, nitrogen, and phosphorus in tree, shrub and herb organs and those in soil and litter; (D, E, F) showed correlation between the stoichiometric ratio of C:N, C:P, N:P in tree, shrub, herb organs and those in soil and litter. Note: *p < 0.05, **p < 0.01, ***p < 0.001; purple indicates positive correlation and yellow indicates negative. L, leaf; B, branch; S, stem; TB, bark; R, root; G, litter; C, carbon concentration; N, nitrogen concentration; P, phosphorus concentration; C:N, the ratio of carbon to nitrogen; N:P, the ratio of nitrogen to phosphorus; C:P, the ratio of carbon to phosphorus; SOC, soil organic carbon; STN, soil total nitrogen; STP, soil total phosphorus; SCN, the ratio of soil carbon to soil nitrogen; SCP, the ratio of soil carbon to soil phosphorus; SNP, the ratio of soil nitrogen to soil phosphorus.

The C:N:P stoichiometric ratios of the plant organs responded differently to soil and litter nutrient stoichiometry (Fig. 8). SOC was significantly correlated negatively with C concentration of tree leaves, whereas it was significantly correlated positively with the C concentration of herb leaves (Figs. 8A and 8C). N concentration in tree organs was significantly correlated positively with N concentration in litter and soil (except branches), while P concentration in tree leaves and branches was significantly correlated positively with P concentration in litter and soil (Fig. 8A). P in herb root was significantly correlated positively with P concentration in litter and soil (Fig. 8C). No clear relationship was observed between shrub nutrients and these in litter and soil (Fig. 8B). The C:N ratios in tree organs were significantly correlated positively with that in litter and soil, while C:P was only significantly correlated positively with that in litter (Fig. 8D). N:P ratio of shrub leaves was significantly correlated positively with that in litter, and C:P of herb roots was significantly correlated positively with that in litter (Figs. 8E and 8F). The significant positive correlations between the litter and soil were linked by their N and P concentrations and C:N ratios (Fig. 8).

## Discussion

### C:N:P stoichiometric characteristics in ecosystem components

For all trees, the mean leaf C, N and P contents were 484.26, 15.6 and 1.86 mg g^−1^ respectively. Leaf N concentration was lower, while P concentration was higher than those of China’s terrestrial plants (18.6, 1.21 mg g^−1^) or the global flora (20.1, 1.77 mg g^−1^) (Han et al., 2005; Reich & Oleksyn, 2004). The mean leaf C of the trees was higher than that of the grassland biomes of China (438 mg g^−1^) and of global flora (461 mg g^−1^) (Elser et al., 2000; He et al., 2006). Significant differences in C:N:P stoichiometry were detected in plant organs in all forest types (Figs. 2 and 3). Due to genetic and evolutionary differences, plants can adjust their demand for specific nutrient elements (Gong et al., 2017), which consequently results in C:N:P stoichiometric differences between plant organs (Sistla & Schimel, 2012). Different plant functional groups (tree, shrub and herb) have a common set of rules that allocate more N and P in leaves (although P is not significantly higher in shrub leaves) and have a higher N:P ratio in leaves than in other organs (Figs. 2–3B, 3C and 3F). This finding aligns with previous studies showed that plant leaves had higher nutrient concentrations than non-leaf organs (Hong, Wang & Wu, 2014; Zhang et al., 2018c). Leaves are responsible for many physiological functions (e.g., photosynthesis, transpiration and respiration) and require higher quantities of N and P to complete diverse biochemical processes (Minden, Kleyer & Byers, 2014). Furthermore, Leaves can maintain a relatively constant higher N:P ratio to meet the physiological needs of metabolic processes, while other organs, with P concentrations rising faster than N concentrations, have a lower N:P ratio (Kerkhoff et al., 2006; Zhang et al., 2018a).

Tree organs in BM had general higher N and P concentrations and general lower C concentrations, C:N ratios and C:P ratios than those in CM (Figs. 2A–2E). These findings correspond with those of Cao & Chen (2017) and Han et al. (2005), who reported higher C concentration, C:N ratio and C:P ratio in coniferous than in deciduous species and higher N and P concentrations in deciduous than evergreen species. Firstly, conifers have many kinds of structural carbohydrates (C-rich), such as lignin, tannins and waxes, and lower N and P contents, resulting in higher C concentrations, C:N ratios and C:P ratios (Thomas & Martin, 2012). Secondly, the higher C:N and C:P ratios reflect higher plant N and P use efficiency (Ge & Xie, 2017). Coniferous species are often confined to nutrient-limited habitats (Aerts & Chapin, 1999), but still maintain the accumulation and increase of biomass. Thus, the coniferous species have a higher N and P utilization efficiency, leading to higher C:N and C:P ratios. Moreover, a previous study proposed that the nutrient supply status can determine the nutrient concentrations in plant organs (He et al., 2008). In our study, the soil N and P concentrations were higher in BM than in the other forest types (Figs. 4B and 4C), which may have caused higher N and P contents and lower C:N and C:P ratios in organs in BM than in BCM and CM. In contrast, the C, N and P concentrations and stoichiometric ratios of understory plants were also significantly different among the forest types, but the concentrations were different in different organs, with no consistent pattern among forest type (Fig. 3). A possible explanation for these results may be that different plant functional groups show some degree of below-ground niche partitioning and have different root depth distributions (Büttner & Leuschner, 1994), leading to understory plants having different nutrient utilization strategies from trees, ultimately forming diverse nutrient characteristics patterns.

In the present study, the litter had similar C:N:P stoichiometric characteristics with the tree (Fig. 3). The findings extend that of Megan, Tanguy & Lars (2004), confirming that litter stoichiometric characteristics were generally aligned with those of plants (Megan, Tanguy & Lars, 2004). A possible explanation was that BM had higher soil N and P concentrations than the other two forest types, causing organs higher N and P contents and lower C:N and C:P ratios of plant organs in BM, and further influenced the litter’s stoichiometric characteristics (Wood, Lawrence & Clark, 2006). Simultaneously, coniferous species have higher nutrient utilization efficiency than broadleaf species, with the leaves reabsorbing more nutrients before they fall, resulting in higher C:N and C:P ratios and lower N and P concentrations (Ericsson, 1994). Moreover, trees can produce more litter biomass than understory species annually (Liu et al., 2018) and may have dominated the nutrient characteristics of litter.

In the present study, topsoil (0–20 cm) had significantly higher C, N and P concentrations and ratios of C:P and N:P than subsoil (40–60 cm) (Figs. 4A–4C, 4E and 4F). This result is in general agreement with the results of previous studies conducted in forest and grassland systems (Prusty, Chandra & Azeez, 2009; Yang & Chen, 2017). A possible explanation for the result is that topsoil nutrients are mainly affected by the return surface litter and soil microorganisms (Jobbagy & Jackson, 2000). With increasing soil depth, the input of organic matter is limited by the permeability of the soil, and microbial decomposition activity gradually decreases (Berger, Neubauer & Glatzel, 2002), leading to the striking stratification characteristics of soil nutrients. Among the different forest types, the soil in CM had generally lower C, N and P concentrations and ratios of C:P and N:P than BM and BCM (Figs. 4A–4C, 4E and 4F). This result may be explained by the fact that litter in BM and BCM had relatively higher N and P concentrations (Figs. 3B and 3C), which can better stimulate microbial activity and invertebrate digestion (Kerkhoff et al., 2006), ultimately benefiting litter decomposition and promoting soil nutrient accumulation. Furthermore, the litter biomass in BM and BCM was notably lower than that in CM (Table S2), which also supported this explanation. In comparison, the C:N ratio was nonsignificant among the different soil layers and in different forest types (Fig. 4D), which may be due to the close temporal coupling of C and N contents in the litter decomposition process, which is consistent with the conclusion from a secondary forest study (Yang & Luo, 2011). In general, these results suggested that the content of soil N and P may be attributable to the forest type (Jerabkova, Prescott & Kishchuk, 2006).

### C, N and P nutrient stock in ecosystem

The highest C stock was observed in the stems of trees, and the highest C, N and P stock values were observed in the roots of shrubs (except for N in BCM) and leaves of herbs (Figs. 5–7A and 7B). We can explain these findings by the higher levels of biomass in these plant organs (Figs. S1A and S1B) and the relatively higher nutrient concentration (Figs. 2 and 3A–3C) (Peichl & Arain, 2006; Yu et al., 2015). However, the highest N and P stock values in trees was not in the stem, which had the highest biomass (except for P in CM); rather, the highest values were generally in branches (Figs. 6–7A). This result corresponds with the results of Frédéric, Mathieu & Quentin (2010), who reported that the contribution of stem wood to total nutrient stock was generally lower than its contribution to total biomass. Among the different forest types, the nutrient stock of different organs and the total biomass nutrient stock were significantly different in the vegetation layers (Figs. 5–7A and 7B; Tables 2–4). This result is most likely associated with the diversity of species composition, biomass and nutrient concentration, which together determined the nutrient stock in the plant organs and different vegetation layers (Frédéric, Mathieu & Quentin, 2010; Gong et al., 2017).

Our study suggested that C, N and P stock in litter biomass in CM generally exceeded that in the BM and BCM (Tables 2–4). This finding agrees with previous studies found that, compared with broadleaf tree species, conifers tend to store a relatively higher amounts of nutrient elements in a labile litter layer (Cremer, Kern & Prietzel, 2016). Because conifer litter had higher lignin and C/N ratios and lower Ca concentrations than broadleaf trees, litter decomposition and nutrient release were hampered in conifer forest (Hobbie et al., 2006). The stock of C and N in the topsoil was significantly higher than that in the subsoil because of the addition of litter fall from the more diverse canopy of trees and understory to the surface soil (Kassa et al., 2017). In contrast, the P stock was nonsignificant among the different soil layers (Fig. 7C). Soil P mainly comes from the weathering of soil rock parent material, which is a very slow process, thereby leading to relatively stable P stock under different soil layers (Tian et al., 2010). Nutrient element stock in different soil layers in BM was generally higher than that in BCM and CM (Figs. 5–7C). This result matches the previous conclusions that the annual litter biomass of aboveground and underground components in broadleaf forest is higher than that in coniferous forest (Finer et al., 2007; Li et al., 2005), and the broadleaf forest have more decomposable components and soil biological activity (Augusto et al., 2015), which enhances the soil C, N and P stock. In total, these results indicate divergent forest nutrient conservation strategies, in which CM share more nutrients stored in the labile litter layer and BM share more nutrients stored in the stable soil layer.

In this study, the N and P stock in the plant layers were 0.746–0.486 and 0.180–0.095 t·ha^−1^ respectively, larger than those of China’s mangrove forest (Li, 1997). And C stock in the plant layer was 73.75–84.78 t·ha^−1^, also larger than that in Asia temperate conifer forests (Thurner et al., 2014). These higher level plant nutrient stock indicated the strong resilience of these secondary forests. But, the nutrient stock of the soil layer and the whole ecosystem were generally lower than those of the forests on the Loess Plateau in China and other forest around the world (Cao et al., 2016; Lilienfein & Wilcke, 2003), which implying potential enormous nutrient accumulation. Net ecosystem nutrient element stock in BM was generally higher than that in BCM and CM but with nonsignificant differences (Tables 2–4). This result agrees with the conclusion drawn from a previous study, in which the stock of the C, N and N elements in the coniferous forest was generally lower than that of deciduous species (Cao et al., 2016). However, for nonsignificant differences, this result may be because the community is in the initial stage of succession and has lower nutrient stock in aboveground organism components in our study area (Jiang, Chen & Cao, 2017).

### Connections of C:N:P stoichiometric among ecosystem components

Plant, litter and soil are closely linked and interact with each other in nature ecosystems; however, few examples have been reported to show how the concentrations of C, N and P in litter and soil were related to their concentrations in multiple organs of plants (Zhang et al., 2018a). Our results show that C concentration in tree leaves was significantly positively correlated with SOC; however, C concentration in herb leaves was significantly correlated positively with SOC. Previous study reported that the proper C:N ratio (closed to 25) can promote microbial metabolism and the accumulation of soil nutrients (Mooshammer et al., 2014). In the present study, tree leaves had generally higher C:N ratio (>25) and herb leaves had proper C:N ratio (Fig. S2). Thus, tree leaves may inhibit microbial metabolism and the accumulation of SOC, and herb leaves stimulated SOC accumulation, leading to a negative correlation between SOC and C content in tree leaves, and a positive correlation between C content in herbal leaves. The connection between tree organs and soil (linked by N, P and C:N) is different from that between herbs and soil (linked by P), indicating that the strategy of nutrient utilization varied by plant functional groups (Zhang et al., 2019). However, there was no obvious correlation between nutrients of shrub and these in soil and litter. A possible explanation is that shrubs are often passively disturbed by herbivores in our study area, causing changes in the nutrient status of the shrubs, which eventually leads to decouple of the cycling of shrub nutrients with soil nutrients. Earlier study reported that the decoupling of nutrient cycling relationships among different components of the ecosystem can be observed when plants respond passively to external environmental conditions (Ladanai, Ågren & Olsson, 2010), supported our result. The strong stoichiometric relationship between vegetation and litter, litter and soil were consistent with previous studies (Zhang et al., 2017), likely because a large proportion of the nutrients in the litter came from plant nutrients and then would be released into the soil, and finally used by vegetation. Overall, our results suggested that nutrient concentrations and stoichiometry in multiple plant organs, litter and soil are tightly linked in forest ecosystem.

## Conclusions

Our study suggests that nutrient stoichiometric ratios and nutrient stock were significantly different for different components and the elements of C, N and P are tightly coupled between the plants, litters and soils in the secondary mixed forest ecosystems. All plants allocated the more N and P to leaves. The content of soil N and P may be related to the forest type due to vegetation nutrient concentration difference. BM has more advantages in terms of C, N and P nutrient stock than do BCM and CM in the secondary succession community. The differences correlation between multi-plant organs, litter and soil indicate that different plant functional groups have diverse strategy of nutrient utilization. Collectively, our findings provide valuable data for forest nutrient element stock management and establishing a nutrient cycle model.

## Supplemental Information

10.7717/peerj.9274/supp-1 Supplemental Information 1 Tree and understory plant organ biomass (A, B) and soil BD (C) in three secondary mixed forests. Different lowercase letters above the bars indicate significant differences among different forest types for the same organ or soil layer (*p* < 0.05), while different uppercase letters indicate significant differences among different organs or soil layers for the same forest type (*p* < 0.05). SL, shrub leaf; SB, shrub branch; SR, shrub root; HA, herb aboveground; HU, herb underground; BM, broadleaf mixed forests; BCM, broadleaf-conifer mixed forests; CM, coniferous mixed forests; BD, bulk density.

10.7717/peerj.9274/supp-2 Supplemental Information 2 The mean C:N ratios of tree leaves and herbleaves.

10.7717/peerj.9274/supp-3 Supplemental Information 3 Allometric equations used for biomass calculation. W: the dry biomass (kg) of a tree component (e.g. stem, bark, branch, leaf and root), D: diameter at breast height for tree (cm), H: height of tree (m), R: Correlation coefficient.

10.7717/peerj.9274/supp-4 Supplemental Information 4 Total biomass of plant layers, ecosystem plants and litter. Different letters indicate significant differences (*p* < 0.05) among forest types based on a one-way ANOVA followed by an LSD test. BM: broadleaf mixed forests, BCM: broadleaf-conifer mixed forests, CM: coniferous mixed forests.

10.7717/peerj.9274/supp-5 Supplemental Information 5 Raw data: species, characteristics of plots, biomass, plant nutrient concentrations and soil attributes.

## Additional Information and Declarations

### Data Availability

The following information was supplied regarding data availability: The raw measurements are available as a Supplemental File.
