# Supplementary figures and images for: The linkages of plant, litter and soil C:N:P stoichiometry and nutrient stock in different secondary mixed forest types in the Qinling Mountains, China
Source: PeerJ. 2020 Jun 3;8:e9274. doi: 10.7717/peerj.9274 (PMC7275688; doi:10.7717/peerj.9274)

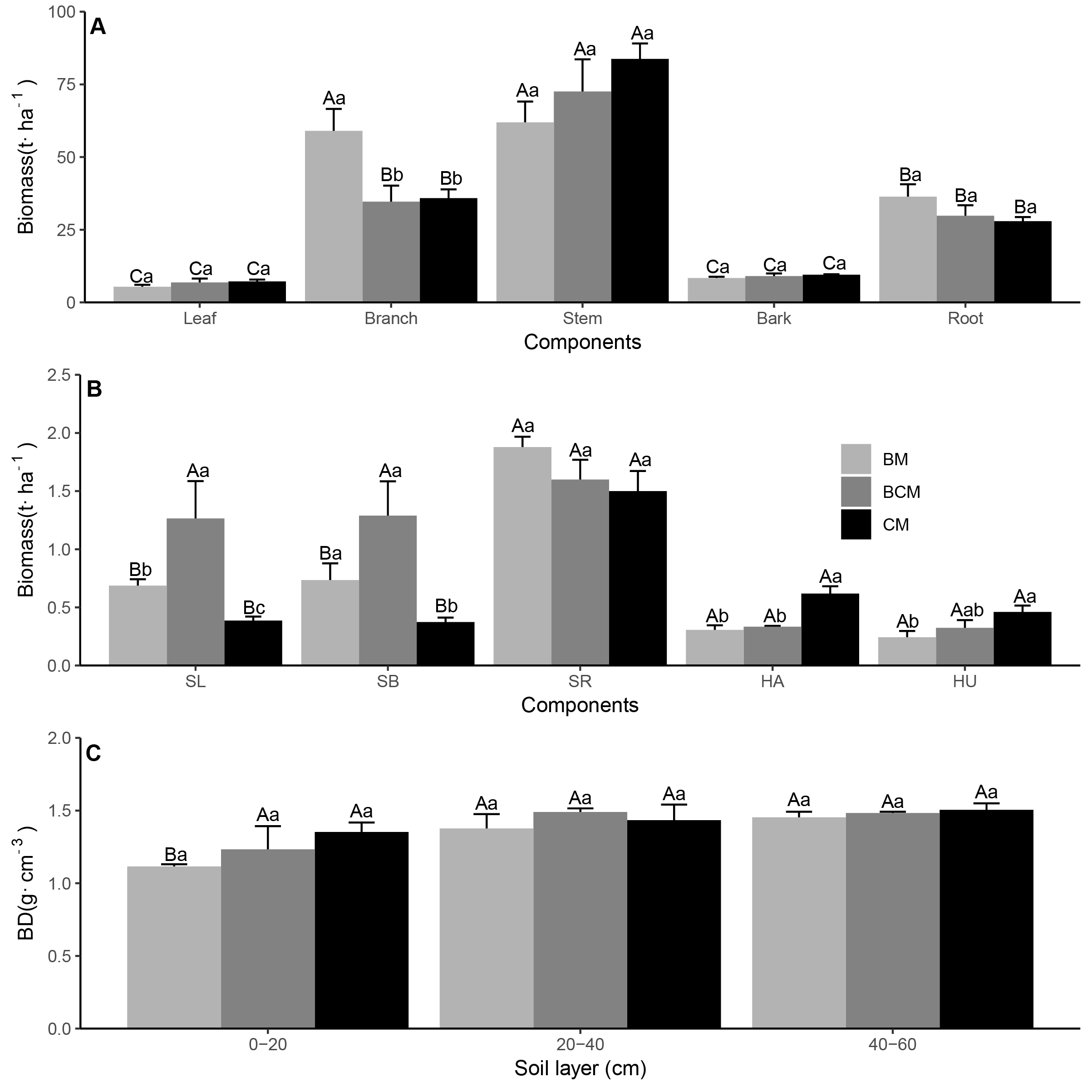

Supplement: Supplemental Information 1 — Different lowercase letters above the bars indicate significant differences among different forest types for the same organ or soil layer (p < 0.05), while different uppercase letters indicate significant differences among different organs or soil layers for the same forest type (p < 0.05). SL, shrub leaf; SB, shrub branch; SR, shrub root; HA, herb aboveground; HU, herb underground; BM, broadleaf mixed forests; BCM, broadleaf-conifer mixed forests; CM, coniferous mixed forests; BD, bulk density. [file peerj-08-9274-s001.png]

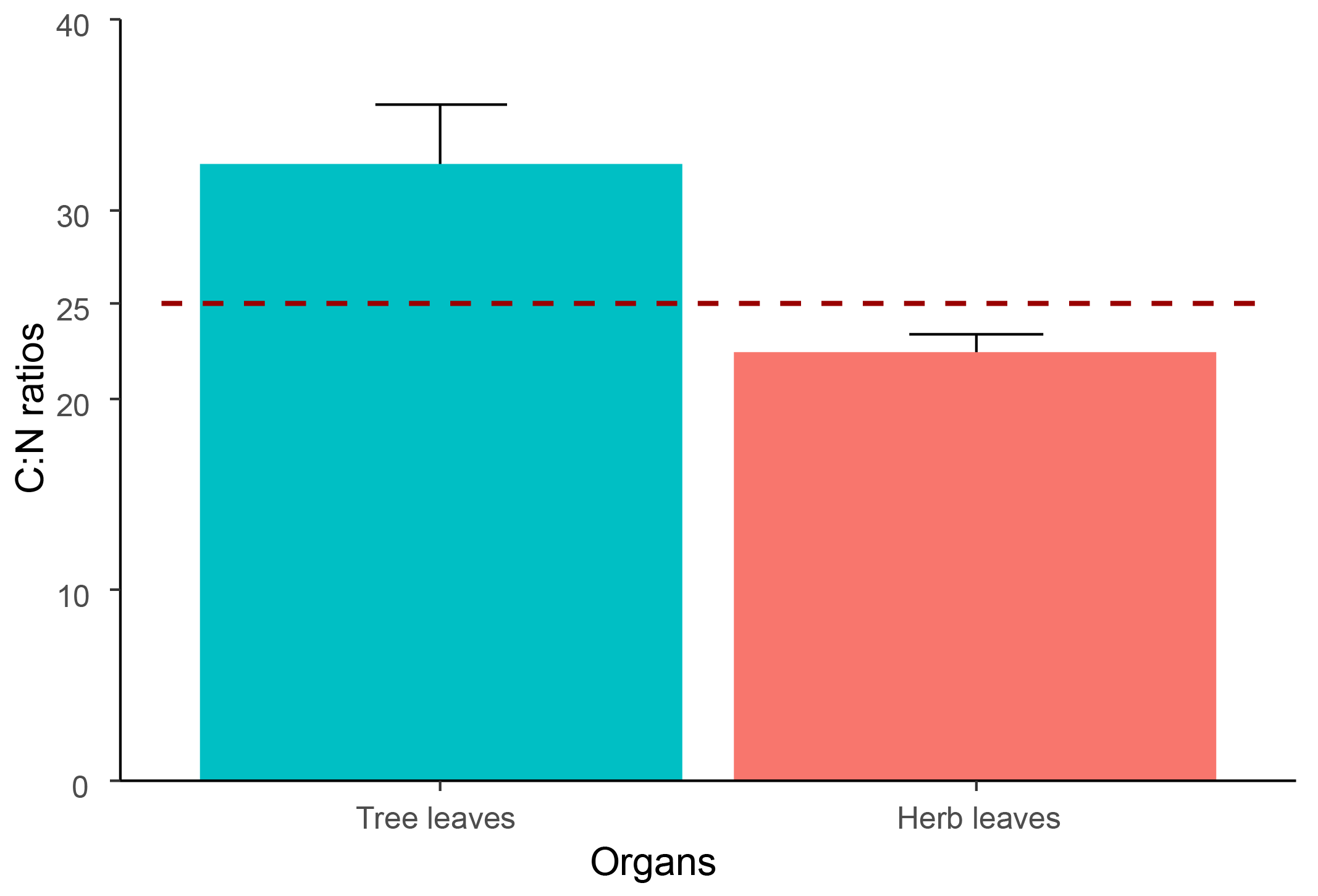

Supplement: Supplemental Information 2 [file peerj-08-9274-s002.png]
