# Supplementary material for: The linkages of plant, litter and soil C:N:P stoichiometry and nutrient stock in different secondary mixed forest types in the Qinling Mountains, China
Source: PeerJ. 2020 Jun 3;8:e9274. doi: 10.7717/peerj.9274 (PMC7275688; doi:10.7717/peerj.9274)
Supplement: Supplemental Information 3 — W: the dry biomass (kg) of a tree component (e.g. stem, bark, branch, leaf and root), D: diameter at breast height for tree (cm), H: height of tree (m), R: Correlation coefficient. [file peerj-08-9274-s003.docx]

| **Species** | **Organ** | **Allometric biomass equation** | **R** |
| --- | --- | --- | --- |
| *Ouercus aliena var. auteserrata* | Stem | lnW_S_=0.99253ln(D^2^H)-3.78818 | 0.99763 |
| *Ouercus aliena var. auteserrata* | Bark | lnW_BA_=0.75632(D^2^H)-3.9245 | 0.99708 |
| *Ouercus aliena var. auteserrata* | Branches | lnW_B_=3.49934lnD-6.50726 | 0.96524 |
| *Ouercus aliena var. auteserrata* | Leaf | lnW_L_=2.29344InD-4.88581 | 0.97832 |
| *Ouercus aliena var. auteserrata* | Root | lnW_R_=2.76435InD-4.20817 | 0.99106 |
| *Quercus variabilis* | Stem | lnW_S_=0.9679ln(D^2^H)-3.7447 | 0.99558 |
| *Quercus variabilis* | Bark | InW_BA_=0.7156ln(D^2^H)-3.2565 | 0.99037 |
| *Quercus variabilis* | Branches | InW_B_=1.0013ln(D^2^H)-4.8449 | 0.99159 |
| *Quercus variabilis* | Leaf | InW_L_=0.6050ln(D^2^H)-3.3569 | 0.98652 |
| *Quercus variabilis* | Root | lnW_R_=0.8144ln(D^2^H)-2.9066 | 0.98792 |
| *Pinus armandii* | Stem | lnW_S_=1.02363In(D^2^H)-4.49970 | 0.99802 |
| *Pinus armandii* | Bark | InW_BA_=0.88417In(D^2^H)-5.38472 | 0.99698 |
| *Pinus armandii* | Branches | InW_B_=2.57551InD-4.08452 | 0.98656 |
| *Pinus armandii* | Leaf | InW_L_=2.75687InD-5.75891 | 0.98004 |
| *Pinus armandii* | Root | lnW_R_=0.97120In(D^2^H)-5.26301 | 0.97927 |
| *Betula albosinensis* | Stem | lnW_S_=0.91035In(D^2^H)-3.79326 | 0.99721 |
| *Betula albosinensis* | Bark | InW_BA_=0.81021In(D^2^H)-4.27750 | 0.99674 |
| *Betula albosinensis* | Branches | InW_B_=3.35934lnD-5.93511 | 0.98584 |
| *Betula albosinensis* | Leaf | InW_L_=2.39007lnD-5.56930 | 0.98709 |
| *Betula albosinensis* | Root | InW_R_=2.68879InD-4.33607 | 0.99292 |
| *Picea asperata* | Stem | lnW_S_=0.9434ln(D^2^H)-3.9744 | 0.9858 |
| *Picea asperata* | Bark | lnW_BA_=0.893ln(D^2^H)-5.5587 | 0.9855 |
| *Picea asperata* | Branches | lnW_B_=0.9257ln(D^2^H)-4.635 | 0.955 |
| *Picea asperata* | Leaf | lnW_L_=0.9753ln(D^2^H)-5.9391 | 0.9848 |
| *Picea asperata* | Root | lnW_R_=0.9457ln(D^2^H)-5.2791 | 0.9919 |
| *Populus davidiana* | Stem | lnW_S_=0.9631ln(D^2^H)-3.8023 | 0.9919 |
| *Populus davidiana* | Bark | lnW_BA_=0.9682ln(D^2^H)-5.833 | 0.9561 |
| *Populus davidiana* | Branches | lnW_B_=1.0903ln(D^2^H)-5.907 | 0.943 |
| *Populus davidiana* | Leaf | lnW_L_=0.6104ln(D^2^H)-3.9108 | 0.791 |
| *Populus davidiana* | Root | lnW_R_=0.7692ln(D^2^H)-3.2756 | 0.9815 |
| Other broad leaf species | Stem | lnW_S_=0.9803ln(D^2^H)-3.8852 | 0.9844 |
| Other broad leaf species | Bark | lnW_BA_=0.7713ln(D^2^H)-4.448 | 0.914 |
| Other broad leaf species | Branches | lnW_B_=1.0851ln(D^2^H)-5.6360 | 0.8869 |
| Other broad leaf species | Leaf | lnW_L=_0.7377ln(D^2^H)-4.5320 | 0.8705 |
| Other broad leaf species | Root | lnW_R_=0.7758ln(D^2^H)-3.1575 | 0.8282 |
