# Supplementary material for: The linkages of plant, litter and soil C:N:P stoichiometry and nutrient stock in different secondary mixed forest types in the Qinling Mountains, China
Source: PeerJ. 2020 Jun 3;8:e9274. doi: 10.7717/peerj.9274 (PMC7275688; doi:10.7717/peerj.9274)
Supplement: Supplemental Information 4 — Different letters indicate significant differences (p < 0.05) among forest types based on a one-way ANOVA followed by an LSD test. BM: broadleaf mixed forests, BCM: broadleaf-conifer mixed forests, CM: coniferous mixed forests. [file peerj-08-9274-s004.docx]

| **Ecosystem pool** | **BM** | |  | **BCM** | |  | **CM** | |
| --- | --- | --- | --- | --- | --- | --- | --- | --- |
|  | **Biomass** | **percentage** |  | **Biomass** | **percentage** |  | **Biomass** | **percentage** |
| **Trees (t ha^-1^)** | 171.12±17 | 97.8 % |  | 152.99±22 | 96.95% |  | 164.30±7 | 98.00% |
| **Shrub (t ha^-1^)** | 3.3±0.19ab | 1.89% |  | 4.15±0.53a | 2.63% |  | 2.26±0.19b | 1.35% |
| **Herb (t ha^-1^)** | 0.55±0.02b | 0.31% |  | 0.66±0.06b | 0.42% |  | 1.08±0.11a | 0.64% |
| **Ecosystem plant (t ha^-1^)** | 174.97±17 | 100% |  | 157.80±22 | 100% |  | 167.64±7 | 100% |
| **G-litter (t ha^-1^)** | 3.86±0.28b |  |  | 4.21±0.14b |  |  | 5.52±0.22a |  |
